# Supplementary material for: Rapid and Quantitative Detection of Human Antibodies against the 2019 Novel Coronavirus SARS CoV2 and Its Variants as a Result of Vaccination and Infection
Source: Microbiol Spectr. 2021 Sep 29;9(2):e00890-21. doi: 10.1128/Spectrum.00890-21 (PMC8557906; doi:10.1128/Spectrum.00890-21)

SUPPLEMENTARY INFORMATION

ACE2 Competitive Binding Assay Method:

Briefly, 2019 SARS CoV2 RBD, B.1.1.7 RBD, or B.1.351 RBD variants were pre-coated in microtiter plate wells (as received). Diluted blood serum and dilutions of PBS-T eluted dried blood spot samples were then mixed with ACE2 protein and distributed to individual wells. In one study, serum and eluted sample from dried blood samples were serially dilute in a 1:5 dilution series and then subjected to the competitive assay. In the second study, serum and eluted sample from dried blood samples were diluted to the same concentration used for GC-FP and then subjected to the competitive assay. Competitive assay plates were incubated overnight at 4 °C after addition of sample and ACE2 protein. Wells were then washed 4X with 300 µl Ray Biotech wash solution, followed by introduction of an anti-ACE2 labeling antibody. After incubation at RT for 1 hr, wells were again washed 4X with wash solution, followed by introduction of HRP-labeled secondary antibody and incubation for an additional 1 hr. After a final 4X wash step, colorimetric reagent was added and plates were incubated for 30 min at room temp, followed by addition of stop solution and absorbance reading at 450 nm.

Table S1. Subject information for individuals who were infected with COVID-19 or vaccinated with the Pfizer-BioNTech, Moderna, or Johnson & Johnson vaccines. Blood serum samples from acutely infected individuals (hospitalized) were collected during hospitalization, while dried blood spots from infected (non-hospitalized) individuals were collected at least 4 weeks post recovery. For vaccinated individuals, dried blood spots samples were collected at least 2 weeks after the final dose of vaccine.

	Vaccine / Exposure Type	Sample #	Gender	Age	Weeks past 2nd dose	Weeks Since Positive Diagnosis	Sample Type
1	Pfizer	1	M	54	2	n/a	Dried Blood Spot
2	Pfizer	2	M	43	2	n/a	Dried Blood Spot
3	Pfizer	3	F	54	2	n/a	Dried Blood Spot
4	Pfizer	4	M	57	2	n/a	Dried Blood Spot
5	Pfizer	5	M	34	8.5	n/a	Dried Blood Spot
6	Pfizer	6	F	38	4	n/a	Dried Blood Spot
7	Pfizer	7	F	42	2	n/a	Dried Blood Spot
8	Pfizer	8	M	72	2	n/a	Dried Blood Spot
9	Pfizer	9	M	61	2	n/a	Dried Blood Spot
10	Pfizer	10	F	66	2	n/a	Dried Blood Spot
11	Pfizer	11	M	67	2	n/a	Dried Blood Spot
12	Pfizer	12	M	37	4	n/a	Dried Blood Spot
13	Pfizer	13	F	43	4	n/a	Dried Blood Spot
14	Pfizer	14	F	64	2	n/a	Dried Blood Spot
15	Pfizer	15	M	73	3	n/a	Dried Blood Spot
16	Pfizer	16	M	24	2	n/a	Dried Blood Spot
17	Pfizer	17	M	44	2	n/a	Dried Blood Spot
18	Moderna	1	F	69	2	n/a	Dried Blood Spot
19	Moderna	2	M	69	2	n/a	Dried Blood Spot
20	Moderna	3	F	28	5	n/a	Dried Blood Spot
21	Moderna	4	F	57	2	n/a	Dried Blood Spot
22	Moderna	5	F	70	2	n/a	Dried Blood Spot
23	Moderna	6	M	53	2	n/a	Dried Blood Spot
24	Moderna	7	F	33	2	n/a	Dried Blood Spot
25	Moderna	8	M	38	2	n/a	Dried Blood Spot
26	Johnson & Johnson	1	M	65	6.5	n/a	Dried Blood Spot
27	Johnson & Johnson	2	M	42	2	n/a	Dried Blood Spot
28	Johnson & Johnson	3	M	24	2	n/a	Dried Blood Spot
29	Johnson & Johnson	4	M	29	2.5	n/a	Dried Blood Spot
30	Johnson & Johnson	5	M	58	4	n/a	Dried Blood Spot
31	Johnson & Johnson	6	F	19	2	n/a	Dried Blood Spot
32	Johnson & Johnson	7	M	34	3	n/a	Dried Blood Spot
33	Johnson & Johnson	8	F	19	4	n/a	Dried Blood Spot
34	Johnson & Johnson	9	M	33	4	n/a	Dried Blood Spot
35	Previously Infected + Pfizer	1	M	50	5	18	Dried Blood Spot
36	Previously Infected + Pfizer	2	F	49	6	18	Dried Blood Spot
37	Previously Infected + Pfizer	3	M	81	8	7.5	Dried Blood Spot
38	Previously Infected + Moderna	4	F	56	2	7.5	Dried Blood Spot
39	Previously Infected + Pfizer	5	F	18	2	22.5	Dried Blood Spot
40	Previously Infected + Pfizer	6	F	57	1.5	4.5	Dried Blood Spot
41	Previously Infected + Pfizer	7	M	55	2	9	Dried Blood Spot
42	Previously Infected + Moderna	8	F	18	3	20	Dried Blood Spot
43	Previously Infected + Moderna	9	M	68	4	54	Dried Blood Spot
44	Previously Infected - non Hospitalized	1	M	74	n/a	14	Dried Blood Spot
45	Previously Infected - non Hospitalized	2	M	66	n/a	5	Dried Blood Spot
46	Previously Infected - non Hospitalized	3	M	56	n/a	7	Dried Blood Spot
47	Previously Infected - Hospitalized	AR7	De-identified		n/a	<1	Serum
48	Previously Infected - Hospitalized	AR21	De-identified		n/a	<1	Serum
49	Previously Infected - Hospitalized	GS12	De-identified		n/a	<1	Serum
50	Previously Infected - Hospitalized	GS14	De-identified		n/a	<1	Serum
51	Previously Infected - Hospitalized	GS17	De-identified		n/a	<1	Serum

**Total Male**	28
**Total Female**	18
**Median Age**	53.5

Table S2. Subject information (Pfizer-BioNTech vaccinated). Dried blood samples were collected just prior to the 1st dose, at the time of the 2nd dose, and 2 weeks after the 2nd dose.

	**Vaccine / Exposure Type**	**Sample #**	**Gender**	**Age**	**Sample Type**
1	Pfizer	1	M	43	Dried Blood Spot
2	Pfizer	2	M	36	Dried Blood Spot
3	Pfizer	3	F	57	Dried Blood Spot
4	Pfizer	4	F	54	Dried Blood Spot
5	Pfizer	5	M	60	Dried Blood Spot
6	Pfizer	6	F	42	Dried Blood Spot
7	Pfizer	7	M	61	Dried Blood Spot
8	Pfizer	8	M	23	Dried Blood Spot
9	Pfizer	9	F	42	Dried Blood Spot
10	Pfizer	10	M	44	Dried Blood Spot

Figure S1. GC-FP SARS CoV2 antigen chip.

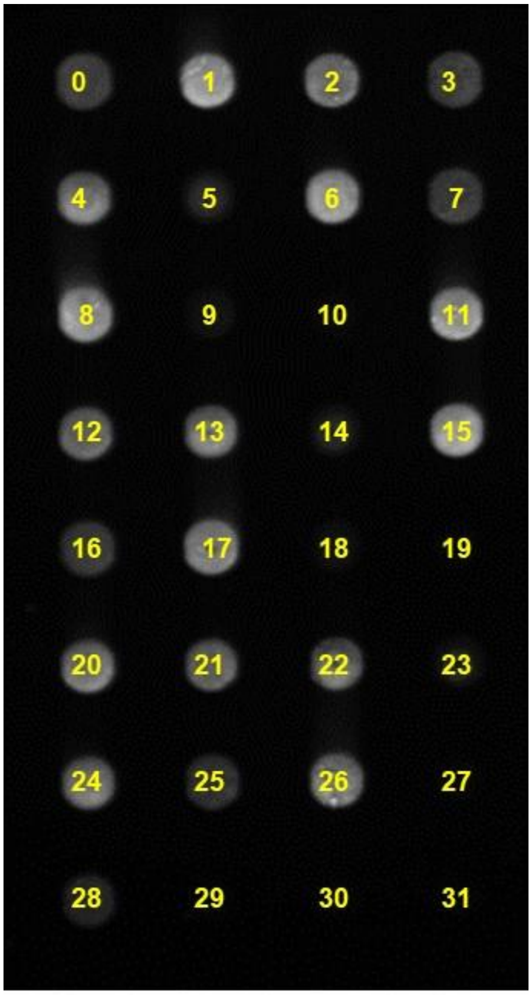

Antigen	Spot Numbers
Human IgG (positive control)	0, 3, 28
SARS CoV2 RBD – B.1.1.7 (N501Y)	1, 11, 20
SARS CoV2 RBD – B.1.351 (K417N, E484K, N501Y)	2, 12, 21
2019 SARS CoV2 RBD	4, 13, 22
2019 SARS CoV2 S1S2+ECD	5, 14, 23
2019 SARS CoV2 S1	6, 15, 24
SARS CoV2 S1 – B.1.351 (K417N, E484K, N501Y, D614G)	7, 16, 25
SARS CoV2 S1 – B.1.1.7 (ΔHV69-70, ΔY144, N501Y, A570D, D614G, P681H)	8, 17, 26
2019 SARS CoV2 Nucleocapsid (N)	9, 18, 27
Human Serum Albumin / HSA (negative control)	10, 19, 29, 30, 31

Figure S2. ~~**A)** Human IgG levels against SARS CoV2 antigens, measured by GC-FP, for vaccinated individuals vs. unvaccinated individuals. The Mann-Whitney test and ROC analysis (see supplementary figure S2) were used to determine statistical significance (*p = 0.03, ** p = 0.002, *** p = 0.0002, **** p < 0.0001).~~ ~~**B)**~~ ROC analysis of human IgG levels (measured by GC-FP) against 2019 SARS CoV2 for vaccinated vs. unvaccinated individuals. Area under the curve (AUC) and the GC-FP diagnostic ratio threshold are reported. The GC-FP diagnostic ratio threshold needed to achieve 100% specificity (false positive rate) is reported, as well as the sensitivity (true positive rate) that can be achieved while maintaining 100% specificity.

~~**A**~~ ~~**B**~~

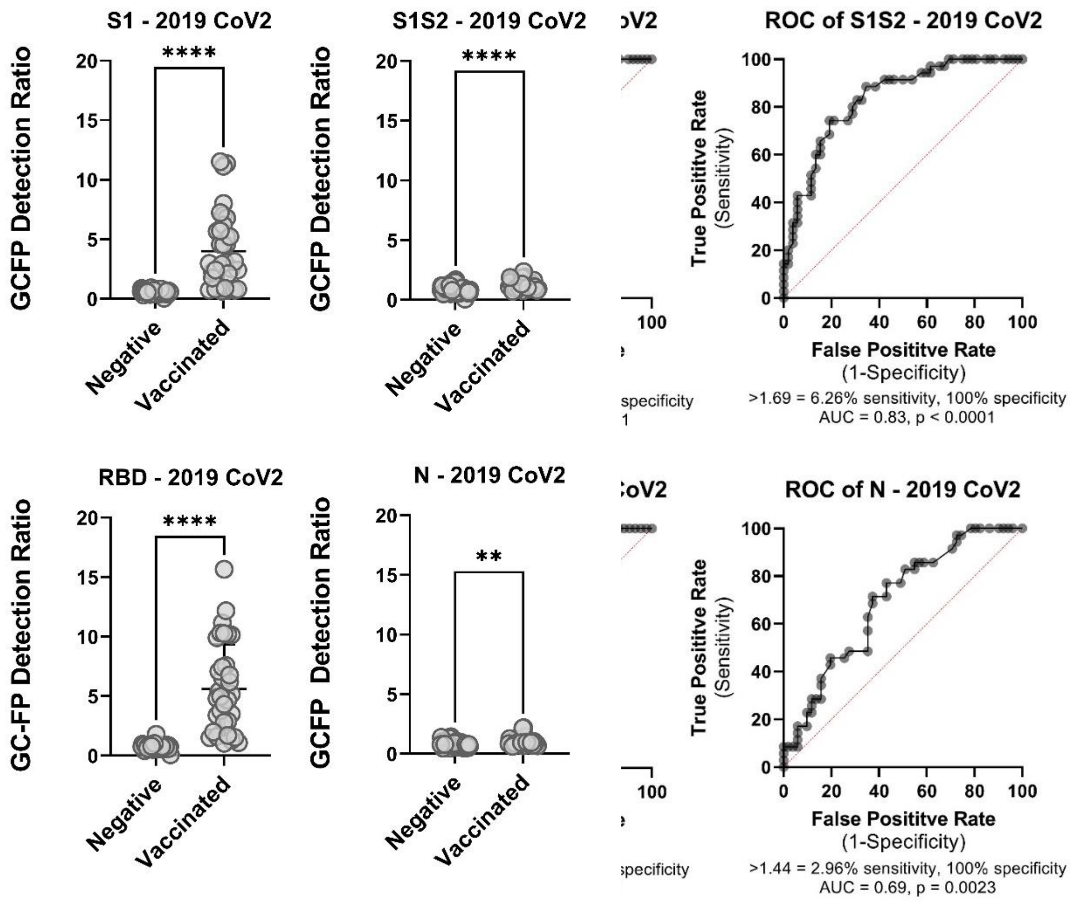

Figure S3. Human IgG levels against SARS CoV2 antigens from the original 2019 SARS CoV2 strain and variant strains B.1.1.7 and B.1.351. GC-FP diagnostic ratio is reported as a measure of IgG level against each antigen, for multiple exposure scenarios and vaccination status (hospitalized and non-hospitalized CoV2 positive, previously CoV2 positive with subsequent vaccination, and at least 2 weeks past vaccination with Pfizer-BioNTech, Moderna or Johnson & Johnson vaccine). One-way ANOVA followed by Dunnett's multiple comparison testing was performed (*p = 0.03, ** p = 0.002, *** p = 0.0002, **** p < 0.0001).

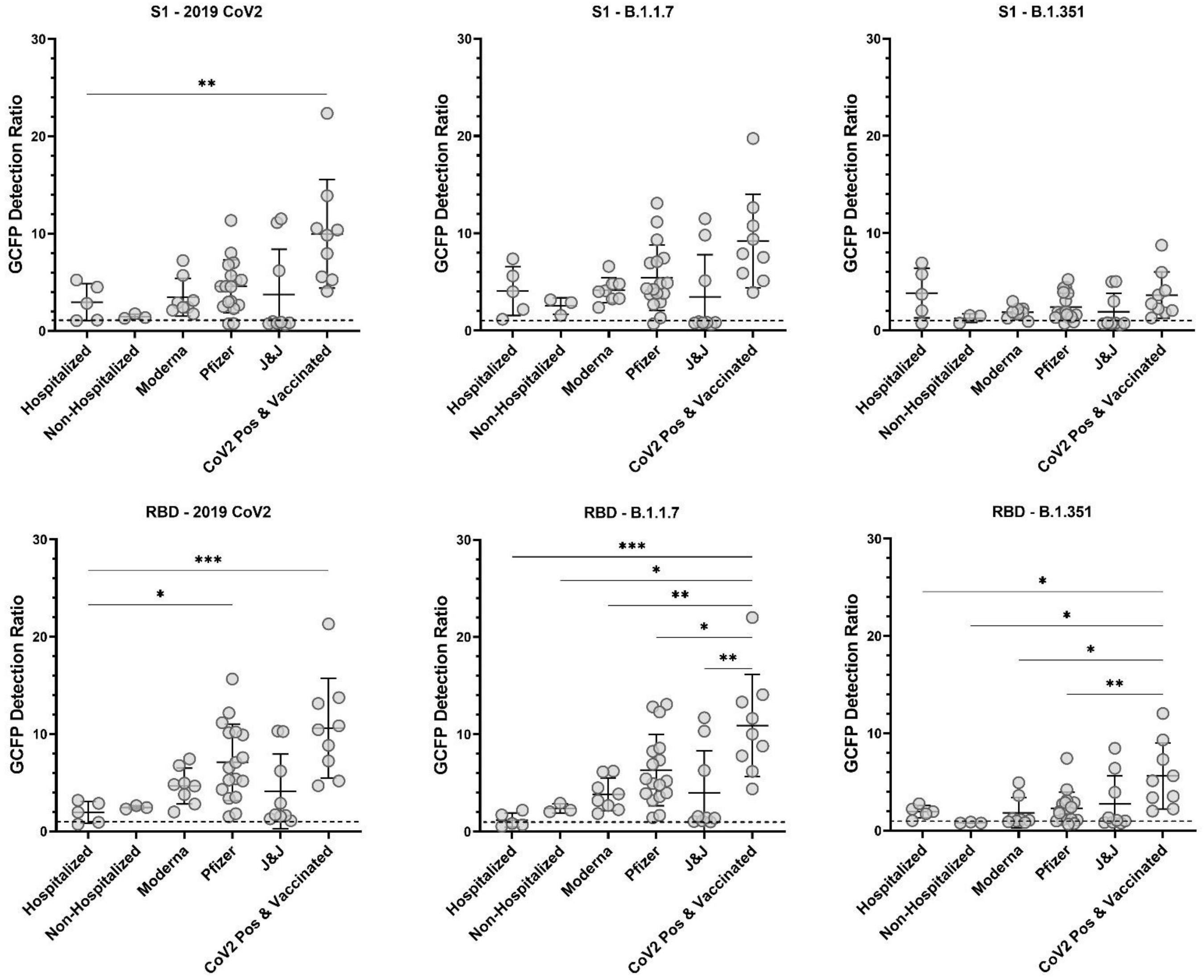

Figure S4. Correlation of the fold change in ACE2 binding inhibition or GC-FP diagnostic ratio for B.1.1.7 and B.1.351 variants of RBD vs. 2019 CoV2 RBD.

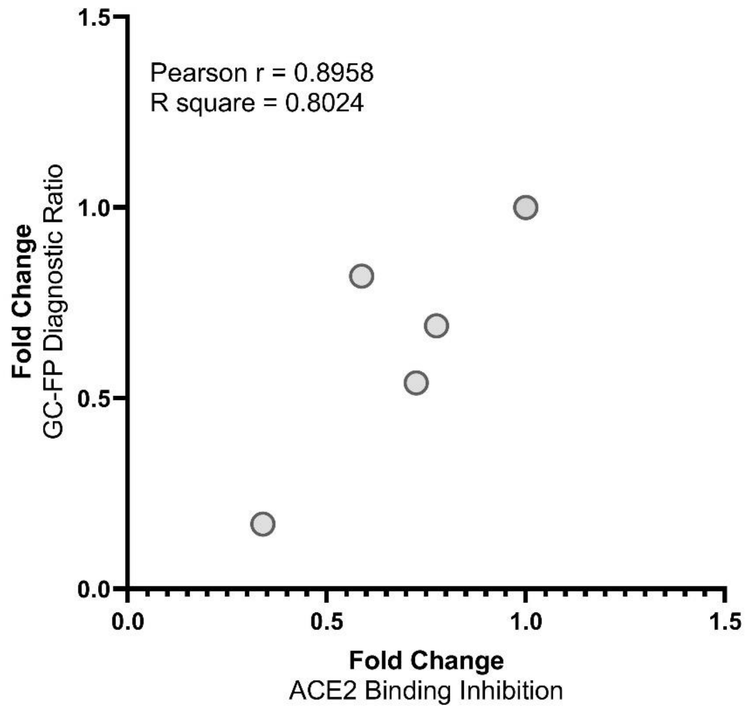

Supplement: SUPPLEMENTAL FILE 1 — Supplemental material. Download SPECTRUM00890-21_Supp_1_seq2.pdf, PDF file, 1.0 MB [file spectrum00890-21_supp_1_seq2.pdf]
